# Kynurenine pathway metabolomics predicts and provides mechanistic insight into multiple sclerosis progression

**DOI:** 10.1038/srep41473

**Published:** 2017-02-03

**Authors:** Chai K. Lim, Ayse Bilgin, David B. Lovejoy, Vanessa Tan, Sonia Bustamante, Bruce V. Taylor, Alban Bessede, Bruce J. Brew, Gilles J. Guillemin

**Affiliations:** 1 Department of Biomedical Sciences, Faculty of Medicine and Health Sciences, Macquarie University, NSW 2109, Australia; 2 Department of Pharmacology, School of Medical Sciences, University of New South Wales, NSW 2052, Australia; 3 Department of Statistics, Faculty of Science and Engineering, Macquarie University, NSW 2109, Australia; 4 Bioanalytical Mass Spectrometry Facility, University of New South Wales, NSW 2052, Australia; 5 Menzies Research Institute Tasmania, University of Tasmania, TAS 7000, Australia; 6 ImmuSmol, Pessac, France; 7 Peter Duncan Neurosciences Research Unit, St Vincent’s Centre for Applied Medical Research, Sydney, Australia; 8 Department of Neurology, St Vincent’s Hospital, Sydney, Australia

## Abstract

Activation of the kynurenine pathway (KP) of tryptophan metabolism results from chronic inflammation and is known to exacerbate progression of neurodegenerative disease. To gain insights into the links between inflammation, the KP and multiple sclerosis (MS) pathogenesis, we investigated the KP metabolomics profile of MS patients. Most significantly, we found aberrant levels of two key KP metabolites, kynurenic acid (KA) and quinolinic acid (QA). The balance between these metabolites is important as it determines overall excitotoxic activity at the N-methyl-D-Aspartate (NMDA) receptor. We also identified that serum KP metabolic signatures in patients can discriminate clinical MS subtypes with high sensitivity and specificity. A C5.0 Decision Tree classification model discriminated the clinical subtypes of MS with a sensitivity of 91%. After validation in another independent cohort, sensitivity was maintained at 85%. Collectively, our studies suggest that abnormalities in the KP may be associated with the switch from early-mild stage MS to debilitating progressive forms of MS and that analysis of KP metabolites in MS patient serum may have application as MS disease biomarkers.

Increasing evidence supports the hypothesis that inflammation contributes to neurodegeneration and is linked to multiple sclerosis (MS) progression^1^. Weiner HL (2009) described how the adaptive immunity mediated by auto-reactive T-cells, fuels the early stages of MS (i.e. relapsing-remitting multiple sclerosis; RRMS)^2^, while monocytic cells of the innate immunity contribute to further neuronal degeneration that exacerbates disease progression leading to secondary progressive MS (SPMS)^2^. However, it is not well understood why approximately 50% of RRMS patients progress to secondary progressive MS, while 50% do not. We sought to better understand the mechanistic drivers of this switch. As the kynurenine pathway (KP) of the tryptophan metabolism is highly inducible in inflammatory environments, we hypothesized that changes in the KP may be associated with the progressive switch in MS.

The kynurenine pathway (KP) is the major route that breaks down tryptophan subsequently leading to the production of NAD^+^. In the presence of pro-inflammatory cytokines^3^,^4^, the KP is induced by activation of its first enzyme, indoleamine 2,3-dioxygenase (IDO-1). Metabolites produced along the KP can have neurotoxic or neuroprotective effects. Quinolinic acid (QA) is perhaps the most important, leading acutely to human neuronal death and chronically to dysfunction by at least 7 separate mechanisms, of which, N-methyl-D-Aspartate (NMDA) receptor excitotoxicity is the best characterised^5^. Within the brain and CNS, QA is produced by activated microglia and infiltrating macrophages but not in neurons or astrocytes^6^. Kynurenic acid (KA), produced by astrocytes^7^, is an antagonist of ionotropic glutamate receptors and thus blocks the excitotoxic effects of QA, KA also has antioxidant activity, readily scavenging hydroxyl, superoxide anion and other free radicals^8^. Indeed, in disease states where excess QA is produced, it is thought that there is insufficient KA to block QA^9^.

Accordingly, we examined the role of the KP in MS progression as it potentially links inflammation-induced activation of the KP^10^, the production of the glutamatergic (NMDA)-modulatory metabolites, KA and QA, to excitotoxic neurodegeneration^11^,^12^,^13^. This metabolic shift may explain why the inflammatory milieu in RRMS changes to a neurodegenerative one in SPMS and may even constitute a unique metabolic biomarker of MS progression. Currently, there are no biomarkers that can identify this transition^14^, and a suitable biomarker would be useful for assessing patient prognosis and potentially new therapeutics.

## Materials and Methods

### Sample Cohorts and Study design

All studies were carried out in accordance with the guidelines of the relevant institutional human research ethics committee and approved by St Vincent’s Hospital Sydney (HREC– H03/037) and Macquarie University (HREC – 5201300333). Our study adheres to ‘The code of ethics of the World Medical Association (Declaration of Helsinki)’ for experiments involving human subjects. Written consent was obtained from respective sources that provided the samples. In order to thoroughly profile KP metabolism in MS, *Cohort 1* samples were obtained through the Accelerated Cure Project for Multiple Sclerosis (ACPMS), USA, and consisted of serum samples from patients with RRMS, SPMS, primary progressive (PPMS) and healthy controls (HC) subjects. Exclusion criteria included MS patients currently receiving disease modifying drugs or who had corticosteroid within the past 3 months, or presence of other medical conditions. Randomized samples were age and gender matched between the experimental groups (i.e. RRMS, SPMS, PPMS and HC) where possible. As repeated blood samples collected over two years were available, *Cohort 2* offered the ability to track changes in the KP longitudinally and was obtained through the Tasmanian MS Longitudinal Study conducted between 2003 and 2005. To further validate our study, we sourced another cohort of MS patients (*Cohort 3*) with matched same-patient serum and cerebrospinal fluid (CSF) from The Human Brain and Spinal Fluid Resource Center (HBSFRC), USA. All samples were unsorted and re-labelled for blinding prior to analysis.

### Profiling of the kynurenine pathway metabolites

All reagents and KP metabolites were analytical reagent grade and were purchased from Sigma-Aldrich (St Louis, MO), unless otherwise stated. Deuterated internal standards were purchased from Medical Isotopes, Inc (Pelham, NH). KP metabolites were extracted using 10% (*w*/*v*) trichloroacetic acid (TCA) with equal volume of serum samples in accordance with methods previously described^15^. CSF samples were prepared similarly to serum samples except that deproteinization with TCA was not performed.

Concurrent analysis of tryptophan, kynurenine, 3-hydroxykynrenine (3-HK), 3-hydroxyanthranilic acid (3-HAA), and anthranilic acid (AA) was performed with UHPLC as described by Jones *et al*.^16^, using an injection volume of 20 μL of the prepared extract from each samples. KA detection was performed using a gradient mobile phase comprise of 50 mM sodium acetate buffer supplement with 25 mM zinc acetate (dihydrate) to enhance fluorescence intensity and 2.25% acetonitrile as organic modifier (Solvent A), and 10% acetonitrile (Solvent B). Each sample (10 μL) was injected into a Poroshell RRHT C-18, 1.8 μm 2.1 × 100 mm column (Agilent Technologies, Inc, Santa Clara, CA) maintained at 38 °C for 12 min run time at a unison flowrate of 0.75 mL/min. The gradient elution consisted of 100% solvent A for 3 min and then 50% solvent A and 50% solvent B for 2 min, followed by 100% B for 2 min and 100% solvent A (run time 10 min). This gradient ensures sufficient time for KA retention while minimizing potential build-up of pressure due to precipitation of the high salt buffer. Detection of KA used fluorescence (excitation and emission wavelengths of 344 and 388 nm, respectively with a retention time of 1.5 min). Agilent OpenLAB CDS ChemStation (Edition C.01.04) was used to analyze the chromatograms (Supplementary Figure S1A and B).

For GCMS, 50 μL of the prepared extract were derivatized. Concurrent analysis of PA and QA were carried out as described by Smythe *et al*.^17^ with slight modification using an Agilent 7890 A GC system coupled with Agilent 5975 C mass spectrometry detector and Agilent 7693 A autosampler (Agilent Technologies, Inc, Santa Clara, CA) with one microliter of derivatized mixture. Separation of PA and QA were achieved with a DB-5MS column, 0.25 μm film thickness, 0.25 mm × 30 m capillary column (Agilent Technologies, Inc, Santa Clara, CA) within 7 min but the assay run time was set for 12 min to prevent sample carryover. Concentrations of PA and QA were analyzed using Agilent GC/MSD ChemStation software (Edition 02.02.1431) and interpolated from the established six-point calibration curves based on the abundance count ratio of the metabolites to their corresponding deuterated internal standards within each standards and samples (Supplementary Figure S1C and D).

The intra- and inter-assay CV was within the acceptable range of 4–8% for UHPLC assays and 7–10% for GCMS assays calculated from the repeated measures of the metabolites standards incorporated during the sequence run. Nicotinamide adenine dinucleotide (NAD^+^) was measured with 20 μL of neat serum using a previously described method^18^.

### Profiling of the inflammatory mediators

Quantification of multiplexing cytokines, chemokines and growth factors was performed using commercial 27-plex magnetic bead based immunoassay kits (Bio-Rad, Hercules, CA) on *Cohort 1*. Each assay was performed in accordance to manufacturer’s instructions at the Australian Proteome Analysis Facility, as described by A. Khan^19^. Final readout of the sample concentration was expressed as picogram per millilitre (pg/mL) based on the standard curves integrated in the assay using the Bio-Plex Manager v5.0 software with reproducible intra- and inter-assay CV of 5–8%.

### Statistical analysis and modelling

The normality of the variables, where required, was checked by Shapiro-Wilk and/or Kolmogorov Smirnov normality tests and equality of the variances by Levene’s test for equality of variances. Comparisons between different MS subtypes and control group were performed by either ANOVA or Kruskal Wallis test, depending on the normality and equal variances analyses. Correlations between variables in *Cohort 1* were assessed by Pearson’s correlations; where required, log transformation of the variables was made before calculating correlations. A *p*-value of <0.05 was considered statistically significant. Paired t-tests or Wilcoxon Signed Ranks tests, depending on the distribution of the variables were applied to investigate the changes to the inflammatory mediators and KP variables from *Cohort 2* where the patients were observed between the years 2003 (baseline, cohort enrollment) and 2005. Four classification methods were used to identify the relative predictive value of each of the variables in each MS subtype in *Cohort 1* (Training set) and then validated against *Cohort 3* (Test set). These methods included the Classification and Regression Tree, Support Vector Machines, Discriminant Analysis and C5.0 Decision Tree^20^,^21^. A classification model was considered successful when it consistently returned high predictive accuracy rates across all three MS subtypes and control group. The model predictions were compared to random predictions (class specific lifts) to decide which model was best for future predictions. In addition, class-specific statistical powers were compared to identify the best classification model. All classification models were developed using IBM SPSS Modeler 14.2, and R^22^ incorporated with various R packages such as Rcmdr, Lawstat, MASS^23^, CAR and Lattice. To investigate whether changes to the CSF KP profiles mirrored serum KP profiles in the controls and MS subtypes of *Cohort 3*, we developed a series of models to identify the best relationships between the CSF KP variables and serum KP variables in the experimental groups. In the first model (model 1), we investigated whether it was possible to predict the CSF value for a KP variable based on its paired serum sample variable. In the second model (model 2), we added the experimental groups as covariates to model 1 to predict the CSF variables. In the last model (model 3), we used model 2 but adjusted for potential confounding factors from all other serum KP variables. We compared these models based on Akaike information criterion (AIC) and adjusted R^2^, to identify the best model(s).

## Results

### Participants’ characteristics

*Cohort 1* made up a total of 136 participants consisting of 50 RRMS, 20 SPMS, 17 PPMS and 49 HC. Age differences between the subtypes were adjusted accordingly in the subsequent analysis. Similarly a total of 59 participants consisting of 44 RRMS and 15 SPMS from *Cohort 2* were included in this study. Each *Cohort 2* participant provided a baseline and follow-up (1.72 years ±0.27) serum samples for analysis. A total of 36 participants from *Cohort 3* met exclusion criteria and provided CSF and matching serum. Accordingly, *Cohort 3* consisted of 10 patients with RRMS, 20 patients with SPMS and 6 HC. Demographic and clinical characteristics of the subjects in all cohorts are summarized in Table 1.

### Activation of the kynurenine pathway in MS

**Figure 1:**
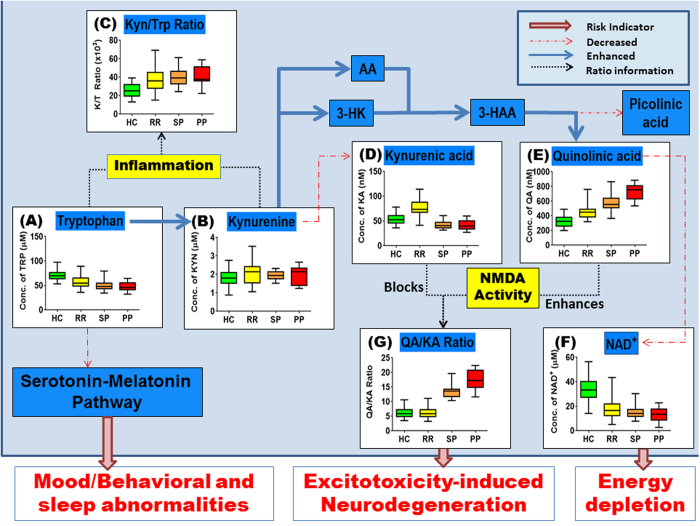
Overview of the kynurenine pathway with box plots of tryptophan (**A**), kynurenine (**B**), kynurenine/tryptophan (Kyn/Trp or K/T) ratio (**C**), kynurenic acid (**D**), quinolinic acid (**E**) NAD^+^ (**F**) and quinolinic acid/kynurenic acid (QA/KA) ratio (**G**) in healthy control (HC), relapsing-remitting MS (RR), secondary progressive MS (SP) and primary progressive MS (PP) cohorts. The diagram illustrate how inflammation can influence the pathway leading to enhance (blue arrows) in some downstream metabolites such as 3-Hydroxykynurenine (3-HK), Anthranilic acid (AA) and 3-Hydroxyanthranilic acid (3-HAA) but not others (red dotted arrows), i.e. kynurenic acid, picolinic acid and nicotinamide adenine dinucleotide (NAD^+^) based on patients with MS when compared to healthy control. The aberrant KP change in MS can potentially lead to mood/behavioural and sleep abnormalities, excitotoxicity-induced neurodegeneration and energy depletion related to cognitive fatigue in MS.

KP activation occurs when the activity of indoleamine 2,3-dioxygenase (IDO-1; the first enzyme in the KP) is significantly higher, leading to the consumption of kynurenine and higher kynurenine/tryptophan (K/T) ratio. In all the MS subtype groups the K/T ratio was significantly increased compared to the HC group (p < 0.0001, Fig. 1A–C; see Supplementary Table S2 for complete KP profile). These data also emphasize the importance of analyzing both TRP and KYN to determine KP activation, as relying on a single metabolite can lead to misinterpretation of data.

### Abnormal downstream KP metabolites production in MS indicating excitotoxicity

As noted above, KA is capable of preventing glutamate-induced excitotoxicity induced by QA. Significantly, we found that KA levels were highest in the RRMS group relative to HC and progressive MS groups (p < 0.0001, Fig. 1D). However, KA levels were significantly lower in the progressive MS groups relative to controls. Picolinic acid, another known neuroprotective KP metabolite^24^, showed a similar trend to KA, being highest in the RRMS but lowest in the PPMS, groups (p < 0.0001, Supplementary Table S2).

Production of QA increased uniformly in concert with disease severity and was particularly elevated in the PPMS group (p < 0.0001, Fig. 1E). We also found decreased NAD+ in all MS patient groups (p < 0.0001, Fig. 1F) which is also indicative of net QA elevation. The QA/KA ratio is indicative of excitotoxic potential with higher QA/KA ratio value favoring excitotoxicity. The QA/KA ratio was higher in both PPMS and SPMS groups compared to controls and the RRMS group (p < 0.0001, Fig. 1G). 3-HK, another potential neurotoxin, was found to be significantly higher in MS groups compared to healthy control (p < 0.0001, Supplementary Table S2). These data support our hypothesis that toxic KP metabolites fuel neurodegeneration in MS.

### KP profile changes involving innate immunity are associated with MS disease severity and subtype

Pearson’s correlation analysis showed that the KP variables correlated significantly to Expanded Disability Status Scale (EDSS) scores suggesting that perturbed KP metabolism may reflect the progression of the disease. Notably, the QA/KA ratio has the strongest correlation with EDSS (r = 0.62, p < 0.0001) underscoring the potential significance of these key KP parameters to the disability and severity of MS (Table 2). Using the Wilcoxon signed rank test for paired data analysis, we showed that the K/T ratio significantly increased over time (p = 0.029) in patients with RRMS demonstrating increased indoleamine 2,3-dioxygenase (IDO-1) activity (Table 3). We also found increased expression of the innate immunity signature proteins, macrophage inflammatory protein (MIP)-1α (p = 0.004) and MIP-1β (p = 0.001) over time in RRMS that are produced by infiltrating macrophages (Table 3). This is accompanied by decrease in adaptive immune respond evident by decreased IL-2 level. Although IL-7 was shown to be significantly increased over time, however, the degree of change is very small (from 13 to 18 pg/ml) and unlikely to be clinically relevant.

### The KP and immune profiles predict the course of disease in MS

**Figure 2:**
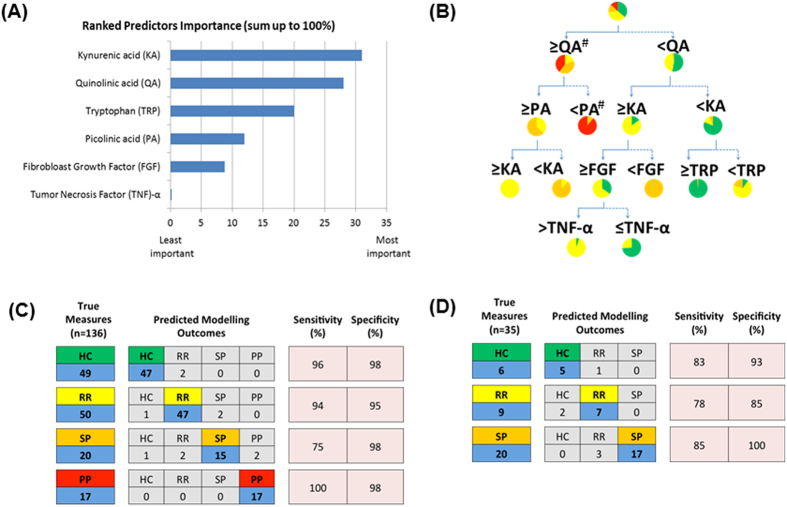
Biomarker for predicting MS severity using in silico approach. (**A**) Classification modelling was based on exploratory analysis on the variables in the dataset with the shortlisted six predictors, i.e., QA, PA, KA, FGF-basic, TRP and TNF-α and its ranked importance for the predictive analytics. (**B**) Training Set using a C5.0 Decision Tree comprised of pie chart proportions of healthy control or MS subtype after being split by the six predictors. To optimize the split, calculated cut-off concentrations for each predictor were determined by the analytic software. The aim is to define a set of predictors that results in a full circle for each experimental group. For example, a QA concentration ≥494 nM (#) results in isolation of the SP and PP MS subtypes, then applying a PA concentration of <313 nM (#), as the next predictor, results in 89.1% isolation of the PP MS subtype. The experimental groups are denoted: healthy control (HC; green), RRMS (RR; yellow), SPMS (SP; orange) and PPMS (PP; red). (**C**) The numbers of observed and correctly predicted HC and MS subtype in the Training Set are shown (blue boxes) along with proportions of true (sensitivity) and false (specificity) predictions. (**D**) A different Test Set was used to validate the predictive model built from the Training Set. The numbers of observed and correctly predicted HC and MS subtype in the Test Set are shown (blue boxes) along with proportions of true (sensitivity) and false (specificity) predictions.

Currently, there are no validated biomarkers of MS^14^ but our data suggests that certain metabolic KP signatures may discriminate MS subtype. To further explore the utility of KP metabolites as MS biomarkers, we applied predictive analytics. Initially, a predictive model was developed incorporating KP metabolites, inflammatory mediators and patient demographic information. The predictive model was then used to yield classification results, i.e., successful identification of MS subtype. From a total of 37 potential predictors, we found six that were the most critical determinants successfully predicting MS subtype (Fig. 2A). These were KA, QA, tryptophan, PA, fibroblast growth factor-basic and tumour necrosis factor-α (in order of relevance). Using various classification models in Training Set, a C5.0 Decision Tree provided the best overall prediction achieving an accuracy of 91% shown in Fig. 2B. We further validated the model using a Test set (*Cohort 3*) and obtained a predictive accuracy of 83% (Fig. 2D). This confirmed that our panel of 6 predictors can be used as a MS subtype biomarker.

To confirm that a facile blood-based KP biomarker for MS is feasible, we correlated CSF KP variables to patient-paired serum profiles (Cohort 3). After adjusting for confounding factors (stratification of clinical grouping and presence of other KP parameters), our regression analysis indicated that serum KP metabolites were able to explain 62.9% (p < 0.001) of changes observed in patient-paired CSF KP variables (Table 4) with a moderately strong (65%) to strong (79.3%) correlation between CSF and serum KP metabolites.

## Discussion

We evaluated the KP in MS in order to explore the links between inflammation, the KP and MS disease progression. Our data indicates that KP metabolism is aberrant in MS, as shown by elevated K/T ratio in MS patients compared to healthy controls, confirming previous studies^25^,^26^. Increased IDO-1 activity (as reflected by higher K/T ratios) is known to suppress the T-cell mediated response in MS^25^,^27^ via activation of aryl hydrocarbon receptor (AhR)^10^. We recently showed that kynurenine, the by-product of IDO1, is an endogenous ligand of the AhR^10^ that inhibits the inflammatory response in chronic experimental autoimmune encephalomyelitis (EAE) mice model of MS^28^. Indeed, inhibition of IDO1 in EAE mice leads to exacerbation of disease progression^29^. Initially, induction of the KP (i.e., up-regulation of IDO-1) may be beneficial as IDO-1 mediates an immunomodulatory effect in MS that partially explains the therapeutic effect exerted by interferons^4^ and vitamin D^30^ in early-mild stages of MS. This is also reflected in our longitudinal data (Cohort 2) with elevated K/T ratio (i.e., increased IDO1 expression) in RRMS but not SPMS to maintain a stable EDSS. However, chronic IDO-1 activation changes the excitotoxic balance due to increased QA production and may also disrupt the biosynthesis of serotonin and melatonin in the brain, as these neurotransmitters are produced in separate branches of the KP that are dependent on tryptophan (Fig. 1). Considering that lower serotonin and melatonin have been associated with depression in MS^31^ and decreased melatonin is known to correlate to increased risk of MS relapse^32^,^33^, restricted tryptophan availability caused by KP activation, may play a role in depression or relapse in MS.

Given that the KP is known to be induced by inflammation, we found surprisingly few correlations between inflammatory mediators and KP modulations. However, a positive correlation was found between interferon (IFN)-g-inducible protein (IP)-10 with K/T ratio (r = 0.31, p < 0.001) and QA (r = 0.2, p < 0.001) indicating an IFN-γ mediated response. Although we did not see significant up-regulation/correlation with IFN-γ and the KP, this may reflect the acute-phase nature of IFN-γ. Considering that the innate immunity markers (MIP-1α and MIP-1β) increased over time in the longitudinal RRMS patient samples (*Cohort 2*), this implies that even with stable EDSS, the innate immune activity is still constitutively active.

Activated innate cells such as macrophages or microglia are known to be the major source of pathophysiological concentrations of QA^34^. We previously showed that oligodendrocytes exposed to QA from activated microglia take up QA leading to oligodendrocyte death. This was reversed by targeting QA production, either by a KP inhibitor or a specific QA targeting antibody^35^. Collectively, our data provides evidence that, over time, the initially suppressive T-cell effect mediated by IDO-1 changes to a more chronic form of KP activation that leads to MS progression by the production of excitotoxic QA (and increased QA/KA ratio) by infiltrating macrophages. Our data support the concept that targeting innate cells may be a feasible immunotherapeutic approach to retard MS disease progression in MS which warrants further investigation.

Increased levels of neuroprotective metabolites, KA and PA, were only observed in RRMS but not in SPMS or PPMS, while toxic metabolites, 3-HK and QA level were progressively increased in both SPMS and PPMS. These observations may imply a role for neurotoxic KP metabolites in mediating neurodegeneration in MS. As described above, QA mediates potent excitotoxicity at the NMDA receptor^36^, whereas KA plays a neuroprotective role as it antagonizes QA excitotoxicity at this receptor. Hence, the balance between QA and KA (expressed as the QA/KA ratio) defines the overall glutamatergic activity at the NMDA receptor and determines whether QA-mediated neurodegenerative excitotoxicity prevails^11^,^12^,^13^. The observed increased level of KA only in RRMS may be a compensatory mechanism in early-stage disease against QA-induced excitotoxicity, as suggested by the moderately strong correlation between QA/KA ratio and MS severity. It also indicates that the KP shunts towards production of KA, but not QA, during early disease course, whereas in later disease stages, the KP is shunted differently, favoring production of QA instead of KA. The mechanism(s) of this differential shunting remains unclear, but delineating these may suggest new therapeutic options. Additionally, as 3-HK is known to potentiate QA-induced excitotoxicity^37^, the higher 3-HK levels in SPMS and PPMS patients observed in our study might also be relevant to the neurodegenerative process in MS. Our results are also consistent with the increased levels of 3-HK and QA found in tissues of the experimental autoimmune encephalomyelitis (EAE) rat model of MS^38^. Considered collectively, our results suggest that NMDA receptor mediated excitotoxicity is highly relevant in the neurodegeneration associated with progressive MS and may constitute a key threshold event in the switch from RRMS to SPMS.

To our knowledge, this is the first study using targeted KP metabolomics as a blood-based prognostic biomarker capable of distinguishing MS subtype. Previously, decreased tryptophan was found in MS patients and described as a potential biomarker^39^. However, this study only detected tryptophan and was not capable of detecting other downstream KP metabolites. This study also failed to distinguish between MS subtype. We showed that tryptophan and 3 other metabolites of the KP were important predictors of MS subtype and correlated to disease severity scores. Indeed, the four KP predictors accounted for approximately 90% of the predictive power of our built model with the two inflammatory mediators only adding 10% predictive power. This suggests that tryptophan metabolism is more relevant to MS pathology than general inflammation. The validity of model was confirmed when applied to an alternate and independent blinded set (Test set, *Cohort 3*) where we observed a reproducible accuracy of 83%.

One potential limitation of a blood based biomarker for neurological disease may be that blood parameters may not necessarily mirror those of the CNS. However, a previous study demonstrated that the blood KP profile followed closely with changes of the KP profile in CNS^40^ and our analysis (which included 3 more KP variables, i.e. K/T ratio, KA and PA) showed generally good to-strong plasma-CNS KP metabolite correlations in the range 65–79.3%. Potential factors limiting these correlations may include the inherent differences in the magnitude of CSF parameters relative to serum values and the fact that the length of storage time (over 10 years) in some of these samples may have led to the partial degradation of some analytes. Although, the key metabolites kynurenine and QA have been reported as relatively stable even after many years of storage^41^. Notwithstanding these points, the moderately strong correlations between CSF and serum, confirms that the serum KP profile is a suitably sensitive blood-based predictor of disease progression in MS.

In conclusion, our results demonstrate that KP parameters have a strong association with MS subtype, correlating with disease severity scores. The changing levels of KP metabolites we observed also provides a mechanistic insight that may explain the transition from the milder RRMS form to the more debilitating SPMS disease form. KP profiling is likely to be relevant to the pathogenesis of other diseases characterized by inflammation and neurodegeneration, like Alzheimer’s disease, Parkinson’s disease and ALS, where aberrant KP metabolism has been reported^42^. Our results also suggest that strategies aimed rebalancing the KP, particularly in terms of QA/KA levels, could be useful therapeutic approaches in slowing neurodegeneration in MS.

## Additional Information

**How to cite this article:** Lim, C. K. *et al*. Kynurenine pathway metabolomics predicts and provides mechanistic insight into multiple sclerosis progression. *Sci. Rep.* **7**, 41473; doi: 10.1038/srep41473 (2017).

**Publisher's note:** Springer Nature remains neutral with regard to jurisdictional claims in published maps and institutional affiliations.

## Supplementary Material

Supplementary Figures

## Figures and Tables

**Table 1:** Demographic and clinical characteristics of Cohort 1, 2 and 3.

	RRMS	SPMS	PPMS	HC
Cohort 1, n	50	20	17	49
Female sex, n(%)	30 (60.0)	15 (75.0)	13 (76.5)	35 (71.4)
Age in years, mean (+SD)^A*^	43.4 (9.4)	53.45 (9.7)	52.24 (8.8)	45.29 (11.7)
Disease duration in year, mean (+SD)^B*^	6.92 (5.9)	16.5 (4.4)	16.18 (5.4)	N/A
Severity, EDSS, median (quartiles)^B*^	2.0 (1.5, 3.4)	6.0 (3.4, 6.5)	5.5 (2.5, 6.0)	N/A
Cohort 2, n	44	15		
Female sex, n(%)	32 (72.7)	7 (46.7)		
Age in years, mean (+SD)^A*^ Baseline 2 years follow-up	47.8 (10.1) 49.4 (10.2)	59.4 (10.7) 61.5 (10.7)		
Disease duration in year, mean (+SD)^B*^ Baseline Follow-up	8.73 (8.4) 10.3 (8.4)	18.2 (9.7) 20.3 (9.9)		
Severity, EDSS, mean (+SD)^B*^ Baseline Follow-up	3.7 (1.9) 3.8 (2.0)	6.8 (1.5) 6.9 (1.5)		
Cohort 3, n	9	20		6
Female sex, n(%)	6 (66.7)	14 (70.0)		4 (66.7)
Age in years, mean (+SD)	49.2 (16.0)	49.7 (9.0)		43.67 (11.8)

Abbreviations: RRMS = Relapsing-remitting multiple sclerosis; SPMS = secondary progressive multiple sclerosis; PPMS = primary progressive multiple sclerosis; HC = healthy controls; EDSS = Expanded Disability Status Scale; N/A = not applicable. SD = Standard deviation. ^A^ANOVA, Tukey HSD, ^B^Kruskal Wallis non-parametric test, *p < 0.001, indicating significant difference between RRMS and SPMS groups. Adjustment had been made accordingly in subsequent analysis.

**Table 2:**
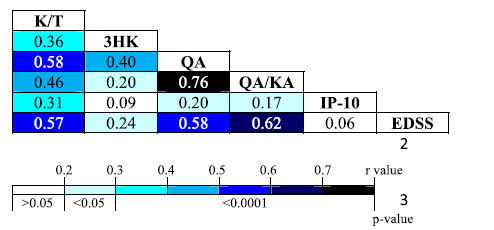
Pearson correlation between KP variables, inflammatory mediators and MS severity (EDSS) scores in Cohort 1.

Table is presented as a heat map, with darker colors depicting the strength of the correlation.All values are expressed as log transformation of the original concentration except K/T Ratio and EDSS.

**Table 3:** KP and Immune profile changes between baseline and 2 years follow-up in Cohort 2 patients with RRMS.

Variables (n)	Baseline, median (quartiles)	Follow-up, median (quartiles)	Fold Change	*p*-value
K/T ratio (44)				
RRMS	54 (40, 65)	57 (47, 71)	0.06	0.029
SPMS	44 (39, 77)	56 (38, 95)	0.27	>0.05
IL-2 (24)				
RRMS	17 (3, 41)	4 (2, 11)	−0.76	0.011
SPMS	9 (2, 16)	3 (1, 7)	−0.67	>0.05
IL-7 (40)				
RRMS	13 (9, 19)	18 (13, 25)	0.38	0.024
SPMS	14 (11, 23)	20 (14, 24)	0.43	>0.05
MIP-1α (28)				
RRMS	5 (4, 10)	10 (5, 13)	1.00	0.004
SPMS	10 (4, 12)	9 (7, 12)	−0.10	>0.05
MIP-1β (41)				
RRMS	37 (21, 71)	69 (48, 90)	0.86	0.001
SPMS	42 (28, 94)	71 (48, 108)	0.69	>0.05

Wilcoxon signed ranks test was use and analyzed by disease group. Other variables measured that are not significant (i.e. p > 0.05) is not listed in the table. No significant changes were observed to any variables in the SPMS group over time. The EDSS was stable with little changes in both RRMS and SPMS shown in Table 1. Concentrations of all the inflammatory mediators are expressed in picogram per mililter (pg/ml).

**Table 4:** Relationship of CSF and Matching Serum KP Profile.

CSF KP variables	Model (#)	Adjusted R^2^	p value	AIC
TRP	1: TRP paired serum only	0.3027	0.0004	51
	***2: TRP paired serum stratified by clinical groups***	***0***.***4442***	***0***.***0003***	***44***
	3: TRP paired serum stratified by clinical groups and adjusted for other KP variables	0.3895	0.0073	52
KYN	1: KYN paired serum only	0.0938	0.0411	−202
	***2: KYN paired serum stratified by clinical groups***	***0***.***5051***	**<*****0***.***0001***	**−*****217***
	3: KYN paired serum stratified by clinical groups and adjusted for other KP variables	0.6142	<0.0001	−225
K/T Ratio	1: K/T Ratio paired serum only	0.2062	0.0036	243
	***2: K*****/*****T Ratio paired serum stratified by clinical groups***	***0***.***5592***	**<*****0***.***0001***	***201***
	3: K/T Ratio paired serum stratified by clinical groups and adjusted for other KP variables	0.4835	0.0008	233
KA	1: KA paired serum only	0.07	0.068	59
	***2: KA paired serum stratified by clinical groups***	***0***.***4234***	***0***.***0004***	***45***
	3: KA paired serum stratified by clinical groups and adjusted for other KP variables	0.3317	0.0178	54
PA	1: PA paired serum only	0.1593	0.01012	271
	***2: PA paired serum stratified by clinical groups***	***0***.***5172***	**<*****0***.***0001***	***229***
	3: PA paired serum stratified by clinical groups and adjusted for other KP variables	0.3548	0.1262	268
QA	1: QA paired serum only	0.334	0.0002	274.8
	***2: QA paired serum stratified by clinical groups***	***0***.***6293***	**<*****0***.***0001***	***215***
	3: QA paired serum stratified by clinical groups and adjusted for other KP variables	0.4592	0.0021	274

Abbreviations: AIC = Akaike information criterion. Note that selection of the best model among the proposed 3 models for each KP metabolites was based on the smallest AIC values. Overall, model 2 across all CSF KP variables has the smallest AIC value and the best model to predict CSF KP variables.
