# Supplementary material for: Kynurenine pathway metabolomics predicts and provides mechanistic insight into multiple sclerosis progression
Source: Sci Rep. 2017 Feb 3;7:41473. doi: 10.1038/srep41473 (PMC5290739; doi:10.1038/srep41473)
Supplement: Supplementary Figures [file srep41473-s1.pdf]

Kynurenine pathway metabolomics predicts and provide mechanistic insight into multiple sclerosis progression

Supplementary Information

Chai K. Lim[1,2*], Ayse Bilgin[3], David B. Lovejoy[1], Vanessa Tan[1], Sonia Bustamante[4], Bruce V. Taylor[5], Alban Bessede[6], Bruce J. Brew[7,8#], Gilles J. Guillemin[1,7#]

*Corresponding author: Chai K. Lim
Department of Biomedical Sciences
Faculty of Medicine and Health Sciences
2, Technology Place, North Ryde
NSW 2109, Australia
Telephone: +61 2 9850 2737
Email: edwin.lim@mq.edu.au

SUPPLEMENTARY FIGURE S1A

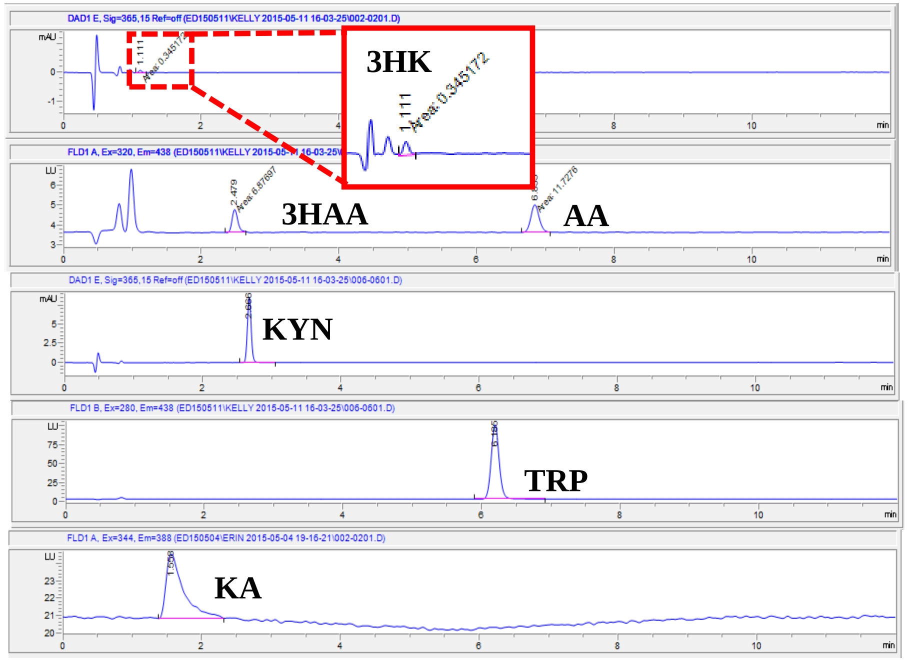

SUPPLEMENTARY FIGURE S1B

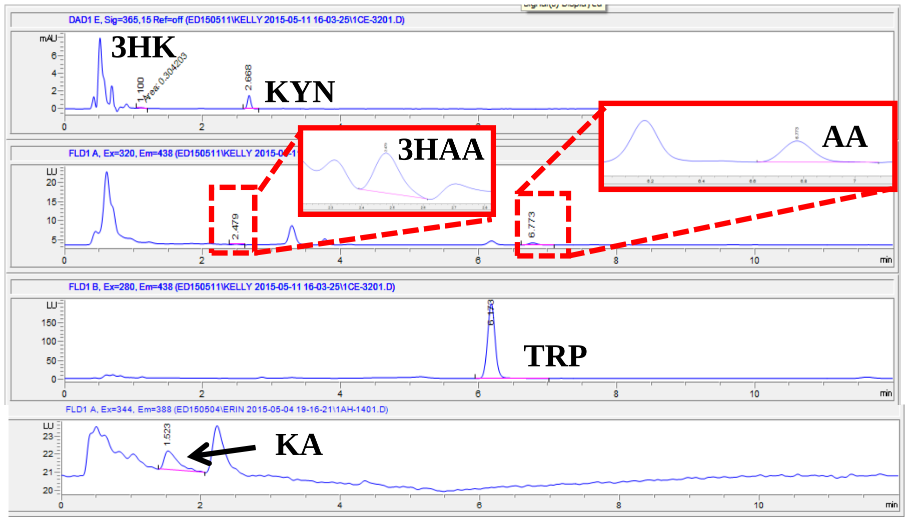

SUPPLEMENTARY FIGURE S1C

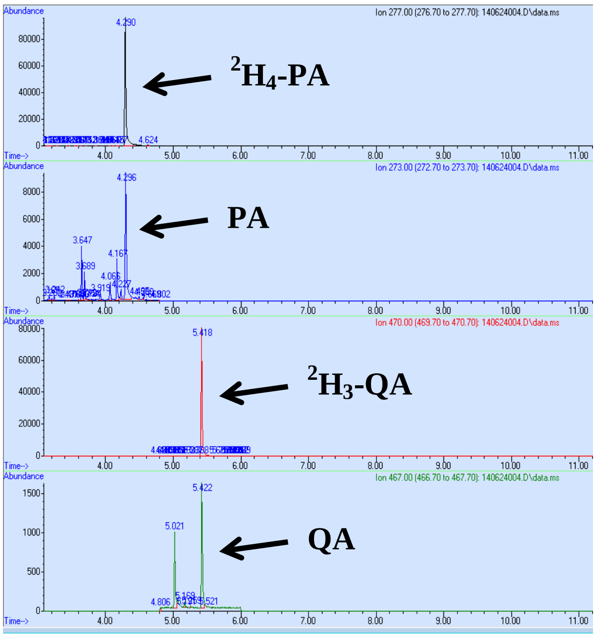

SUPPLEMENTARY FIGURE S1D

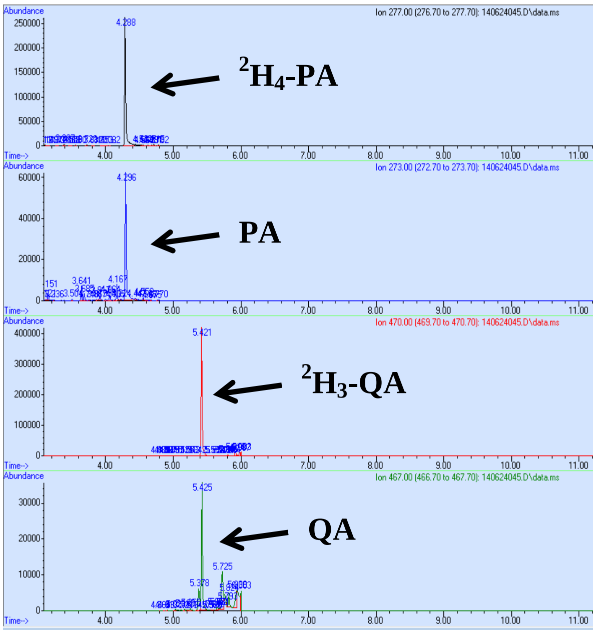

Supplementary Figure 1. Snapshot of chromatograms of various KP metabolites standards (A) and serum sample (B) by UHPLC. Chromatogram of PA and QA standards (C) and serum samples (D) by GCMS. Although 3HAA was measured in our serum samples, the level was abnormally low. 3HAA is a relatively unstable metabolites having limited application as a biomarker and was not shown in our data.

SUPPLEMENTARY TABLES

Supplementary Table S2. Cohort 1 KP Metabolic Profiles

Metabolite	Mean or Median	Standard deviation or IQR	MS versus control Differences	*P value*
**TRP[µM]**				
Control (n=49)	71.11	± 10.20		
RRMS (n=50)	57.99	± 12.83	(D) -13.12	<0.0001[A]
SPMS (n=20)	50.44	± 11.63	(D) -20.67	<0.0001[A]
PPMS (n=17)	47.08	± 8.75	(D) -24.02	<0.0001[A]
**KYN[µM]**				
Control (n=49)	1.81	± 0.41		
RRMS (n=50)	2.05	± 0.51	(U) 0.25	0.03[A]
SPMS (n=20)	1.95	± 0.24		N.S[A]
PPMS (n=17)	1.91	± 0.50		N.S[A]
**K/T Ratio**				
Control (n=49)	26.02	± 7.29		
RRMS (n=50)	36.78	± 11.04	(U) 10.75	<0.0001[A]
SPMS (n=20)	40.12	± 9.09	(U) 14.09	<0.0001[A]
PPMS (n=17)	41.15	± 10.27	(U) 15.13	<0.0001[A]
**KA[nM]**				
Control (n=49)	52.77	46.16 – 60.65		
RRMS (n=50)	73.84	67.48 – 87.42	(U) 22.64	<0.0001[B]
SPMS (n=20)	40.46	36.52 – 46.25	(D) -11.33	0.01[B]
PPMS (n=17)	40.01	34.17 – 49.19	(D) -12.05	<0.01[B]
**3-HK[nM]**				
Control (n=49)	49.15	37.84 – 54.46		
RRMS (n=50)	63.67	48.87 – 81.68	(U) 25.06	<0.0001[B]
SPMS (n=20)	83.01	51.40 – 95.62	(U) 32.13	<0.0001[B]
PPMS (n=17)	83.61	69.46 – 88.92	(U) 33.74	<0.0001[B]
**AA[nM]**				
Control (n=49)	130.02	± 80.83		
RRMS (n=50)	116.73	± 54.35		N.S[A]
SPMS (n=20)	134.42	± 46.57		N.S[A]
PPMS (n=17)	145.54	± 55.36		N.S[A]
**PA[nM]**				
Control (n=49)	392.96	± 77.11		
RRMS (n=50)	459.13	± 91.74	(U) 66.17	<0.001[A]
SPMS (n=20)	388.10	± 66.94		N.S[A]
PPMS (n=17)	246.90	± 51.53	(D) -146.07	<0.0001[A]
**QA[nM]**				
Control (n=49)	322.13	262.26 – 377.54		
RRMS (n=50)	448.97	388.76 – 490.58	(U) 119.97	<0.0001[B]
SPMS (n=20)	552.94	511.34 – 617.71	(U) 240.07	<0.0001[B]
PPMS (n=17)	754.62	629.20 – 803.55	(U) 388.33	<0.0001[B]
**QA/KA Ratio**				
Control (n=49)	5.94	4.88 – 7.15		
RRMS (n=50)	5.88	4.91 – 7.08		N.S[B]
SPMS (n=20)	13.63	11.78 – 14.12	(U) 7.28	<0.0001[B]
PPMS (n=17)	17.23	14.95 – 20.56	(U) 11.41	<0.0001[B]
**NAD^+**				
Control (n=49)	33.54	27.28 – 39.92		

RRMS (n=50)	16.81	12.36 – 21.83	(D) -14.87	<0.0001[B]
SPMS (n=20)	14.20	11.83 – 16.61	(D) -18.32	<0.0001[B]
PPMS (n=17)	13.49	8.63 – 17.13	(D) -20.30	<0.0001[B]

Mean concentration of tryptophan (TRP), kynurenine (KYN), kynurenine/tryptophan (K/T) ratio, kynurenic acid (KA), 3-hydroxykynurenine (3-HK), picolinic acid (PA), quinolinic acid (QA) and QA/KA ratio in stratified MS groups and healthy controls. Metabolites that pass the Shapiro-Wilk normality test ($p \geq 0.05$; data not shown), ANOVA (denoted with A) was used for comparison and reported as mean with standard deviation, otherwise, Kruskal Wallis (denoted as B) was applied and reported as median with interquartile range (IQR) provided. A p value of ≤ 0.05 is considered significant for mean difference between the control and stratified MS subtypes with N.S. denotes as no significant. Further illustration of the mean differences is denote by up (U), and down (D).

Supplementary Table S3. Cohort 1 27-Plex Cytokines, Chemokine and Growth Factor Profiles

**Cytokines, Chemokines and Growth Factors**	**Median**	**IQR**	**MS versus control Differences**$^{\Phi}$	***P value***
**IL-1β**				
Control (n=49)	1.42	1.12-1.74		
RRMS (n=50)	1.15	0.92-1.59		N.S
SPMS (n=20)	1.34	0.95-1.53		N.S
PPMS (n=17)	1.29	1.01-1.69		N.S
**IL-1ra**				
Control (n=49)	69.53	49.65-107.31		
RRMS (n=50)	55.49	37.23-87.09	(D)	N.S
SPMS (n=20)	77.93	49.16-96.47	(U)	N.S
PPMS (n=17)	57.35	48.24-110.38	(D)	N.S
**IL-2**				
Control (n=49)	8.29	5.69-12.02		
RRMS (n=50)	5.54	3.64-9.94	(D)	N.S
SPMS (n=20)	10.21	5.71-12.11	(U)	N.S
PPMS (n=17)	8.99	5.96-12.65		N.S
**IL-4**				
Control (n=49)	1.27	0.97-1.52		
RRMS (n=50)	1.16	0.92-1.37		N.S
SPMS (n=20)	1.17	1.09-1.46		N.S
PPMS (n=17)	1.25	1.04-1.51		N.S
**IL-5**				
Control (n=49)	2.58	1.97-3.08		
RRMS (n=50)	2.14	1.68-2.84		N.S
SPMS (n=20)	2.34	1.90-3.16		N.S
PPMS (n=17)	2.30	2.06-3.12		N.S
**IL-6**				
Control (n=49)	4.68	3.33-6.21		
RRMS (n=50)	3.44	2.48-5.33	(D)	N.S
SPMS (n=20)	5.29	3.22-6.98	(U)	N.S
PPMS (n=17)	4.50	4.17-5.40		N.S
**IL-7**				
Control (n=49)	5.88	3.95-7.92		
RRMS (n=50)	5.26	4.27-8.85		N.S
SPMS (n=20)	6.80	5.57-7.75	(U)	N.S

PPMS (n=17)	6.78	5.65-7.98	(U)	N.S
**IL-8**				
Control (n=49)	13.13	8.73-14.72		
RRMS (n=50)	9.95	8.26-13.06	(D)	N.S
SPMS (n=20)	12.57	10.76-14.36		N.S
PPMS (n=17)	12.98	9.90-13.25		N.S
**IL-9**				
Control (n=49)	9.63	7.10-13.26		
RRMS (n=50)	7.82	6.01-9.78	(D)	N.S
SPMS (n=20)	8.48	6.71-10.84	(D)	N.S
PPMS (n=17)	8.49	7.36-8.49	(D)	N.S
**IL-10**				
Control (n=49)	9.71	6.75-14.76		
RRMS (n=50)	6.49	4.95-10.26	(D)	N.S
SPMS (n=20)	8.09	6.54-12.22	(D)	N.S
PPMS (n=17)	8.79	6.34-12.78	(D)	N.S
**IL-12 (p70)**				
Control (n=49)	20.76	12.13-29.81		
RRMS (n=50)	14.73	10.51-26.75	(D)	N.S
SPMS (n=20)	17.39	11.17-28.48	(D)	N.S
PPMS (n=17)	25.47	13.47-43.14	(U)	N.S
**IL-13**				
Control (n=49)	7.50	5.67-12.71		
RRMS (n=50)	5.55	4.07-10.72	(D)	N.S
SPMS (n=20)	8.68	5.95-12.23	(U)	N.S
PPMS (n=17)	12.02	8.32-15.51	(U)	N.S
**IL-15**				
Control (n=49)	9.77	7.05-16.48		
RRMS (n=50)	9.54	6.84-12.96		N.S
SPMS (n=20)	9.60	7.98-12.30		N.S
PPMS (n=17)	7.83	6.49-10.49	(D)	N.S
**IL-17**				
Control (n=49)	35.34	26.19-45.91		
RRMS (n=50)	28.34	22.72-35.88	(D)	N.S
SPMS (n=20)	32.37	25.60-37.97	(D)	N.S
PPMS (n=17)	30.49	23.52-36.96	(D)	N.S
**Eotaxin**				
Control (n=49)	69.92	42.24-105.27		
RRMS (n=50)	58.19	42.52-89.70	(D)	N.S
SPMS (n=20)	88.98	69.05-137.97	(U)	N.S
PPMS (n=17)	62.06	46.94-95.00	(D)	N.S
**FGF-basic**				
Control (n=49)	14.65	12.34-18.50		
RRMS (n=50)	12.24	9.53-15.33	(D)	N.S
SPMS (n=20)	13.55	9.53-17.89	(D)	N.S
PPMS (n=17)	13.85	10.23-16.96	(D)	N.S
**G-CSF**				
Control (n=49)	101.74	81.24-122.01		
RRMS (n=50)	83.17	70.24-103.33	(D)	N.S
SPMS (n=20)	97.59	80.27-128.77	(D)	N.S
PPMS (n=17)	97.91	85.10-116.96	(D)	N.S
**GM-CSF**				
Control (n=49)	7.64	5.87-12.21		

RRMS (n=50)	5.23	4.03-7.05	(D)	N.S
SPMS (n=20)	4.80	2.34-9.21	(D)	N.S
PPMS (n=17)	6.70	2.03-9.30	(D)	N.S
**IFN-γ**				
Control (n=49)	31.30	24.39-39.38		
RRMS (n=50)	24.83	17.90-32.90	(D)	N.S
SPMS (n=20)	30.12	25.58-37.85	(D)	N.S
PPMS (n=17)	28.72	20.22-33.44	(D)	N.S
**IP-10**				
Control (n=49)	269.58	224.74-465.89		
RRMS (n=50)	296.87	232.79-434.15	(U)	N.S
SPMS (n=20)	379.88	218.55-468.55	(U)	N.S
PPMS (n=17)	324.31	250.42-433.99	(U)	N.S
**MCP-1**				
Control (n=49)	45.35	36.44-65.58		
RRMS (n=50)	33.39	22.46-57.41	(D)	N.S
SPMS (n=20)	47.23	24.62-62.18	(U)	N.S
PPMS (n=17)	45.95	28.91-60-56		N.S
**MIP-1α**				
Control (n=49)	1.75	1.37-2.70		
RRMS (n=50)	1.37	1.00-1.78		N.S
SPMS (n=20)	1.71	1.27-2.03		N.S
PPMS (n=17)	1.69	1.41-2.84		N.S
**MIP-1β**				
Control (n=49)	34.47	26.16-47.07		
RRMS (n=50)	28.01	20.76-41.40	(D)	N.S
SPMS (n=20)	32.17	26.59-39.78	(D)	N.S
PPMS (n=17)	40.23	30.76-54.01	(U)	N.S
**PDGF-bb**				
Control (n=49)	1207.17	966.12-1577.31		
RRMS (n=50)	1193.95	919.28-1611.26		N.S
SPMS (n=20)	1203.98	977.71-1642.65		N.S
PPMS (n=17)	1201.54	1038.91-1380.43		N.S
**RANTES**				
Control (n=49)	1484.55	1269.85-2120.25		
RRMS (n=50)	1469.61	1158.78-1746.00		N.S
SPMS (n=20)	1602.06	1217.05-1602.06	(U)	N.S
PPMS (n=17)	1835.80	1620.58-2440.05	(U)	N.S
**TNF-α**				
Control (n=49)	19.68	14.68-25.83		
RRMS (n=50)	15.79	12.56-21.68	(D)	N.S
SPMS (n=20)	17.18	12.01-22.47	(D)	N.S
PPMS (n=17)	17.45	13.20-25.64	(D)	N.S
**VEGF**				
Control (n=49)	47.58	24.57-70.41		
RRMS (n=50)	33.27	20.52-53.41	(D)	N.S
SPMS (n=20)	32.43	21.74-47.87	(D)	N.S
PPMS (n=17)	44.53	35.57-83.25	(D)	N.S

Median concentration of interleukin-1β (IL-1β), interleukin-1 receptor antagonist (IL-1ra), interleukin-2 (IL-2), interleukin-4 (IL-4), interleukin-5 (IL-5), interleukin-6 (IL-6), interleukin-7 (IL-7), interleukin-8 (IL-8), interleukin-9 (IL-9), interleukin-10 (IL-10), interleukin-12 (p70) [IL-12 (p70)], interleukin-13 (IL-13), interleukin-15 (IL-15), interleukin-17 (IL-17), Eotaxin, Fibroblast growth factor-basic (FGF-basic), granulocyte

colony-stimulating factor (G-CSF), granulocyte-macrophages colony-stimulating factor (GM-CSF), interferon gamma (IFN-γ), IFN-γ-inducible protein 10 (IP-10), monocyte chemotactic protein-1 (MCP-1), macrophages inflammatory protein-1α (MIP-1 α), platelet-derived growth factor (PDGF-bb), macrophages inflammatory protein-1β (MIP-1β), regulated upon activation normal T-cell expressed and secreted (RANTES), tumour necrosis factor-alpha (TNF-α) and vascular endothelial growth factor (VEGF) in stratified MS groups and healthy controls. Kruskal Wallis nonparametric test was used and reported with interquartile range (IQR). We did not see any significance in mean difference between the control and stratified MS subtypes with N.S. denotes as no significant. Further illustration of noticeable mean differences is denote by up (U), and down (D). Concentrations of all the inflammatory mediators are expressed in picogram per mililter (pg/ml).
